# Robust, reproducible, industrialized, standard membrane feeding assay for assessing the transmission blocking activity of vaccines and drugs against *Plasmodium falciparum*

**DOI:** 10.1186/s12936-015-0665-8

**Published:** 2015-04-09

**Authors:** Tao Li, Abraham G Eappen, Adam M Richman, Peter F Billingsley, Yonas Abebe, Minglin Li, Debbie Padilla, Isabel Rodriguez-Barraquer, B Kim Lee Sim, Stephen L Hoffman

**Affiliations:** Sanaria Inc., 9800 Medical Center Drive, Rockville, MD 20850 USA; Protein Potential LLC, 9800 Medical Center Drive, Rockville, MD 20850 USA; Department of Epidemiology, Johns Hopkins Bloomberg School of Public Health, 615 North Wolfe Street, Suite E6003, Baltimore, MD 21205 USA

**Keywords:** *Plasmodium falciparum*, Mosquito, Transmission blocking, Standard membrane feeding assay, Vaccine that interrupts malaria transmission, Gametocyte, Oocyst

## Abstract

**Background:**

A vaccine that interrupts malaria transmission (VIMT) would be a valuable tool for malaria control and elimination. One VIMT approach is to identify sexual erythrocytic and mosquito stage antigens of the malaria parasite that induce immune responses targeted at disrupting parasite development in the mosquito. The standard *Plasmodium falciparum* membrane-feeding assay (SMFA) is used to assess transmission-blocking activity (TBA) of antibodies against candidate immunogens and of drugs targeting the mosquito stages. To develop its *P. falciparum* sporozoite (SPZ) products, Sanaria has industrialized the production of *P. falciparum*-infected *Anopheles stephensi* mosquitoes, incorporating quantitative analyses of oocyst and *P. falciparum* SPZ infections as part of the manufacturing process.

**Methods:**

These capabilities were exploited to develop a robust, reliable, consistent SMFA that was used to assess 188 serum samples from animals immunized with the candidate vaccine immunogen, Pfs25, targeting *P. falciparum* mosquito stages. Seventy-four independent SMFAs were performed. Infection intensity (number of oocysts/mosquito) and infection prevalence (percentage of mosquitoes infected with oocysts) were compared between mosquitoes fed cultured gametocytes plus normal human O^+^ serum (negative control), anti-Pfs25 polyclonal antisera (MRA39 or MRA38, at a final dilution in the blood meal of 1:54 as positive control), and test sera from animals immunized with Pfs25 (at a final dilution in the blood meal of 1:9).

**Results:**

SMFA negative controls consistently yielded high infection intensity (mean = 46.1 oocysts/midgut, range of positives 3.7-135.6) and infection prevalence (mean = 94.2%, range 71.4-100.0) and in positive controls, infection intensity was reduced by 81.6% (anti-Pfs25 MRA39) and 97.0% (anti-Pfs25 MRA38), and infection prevalence was reduced by 12.9 and 63.5%, respectively. A range of TBAs was detected among the 188 test samples assayed in duplicate. Consistent administration of infectious gametocytes to mosquitoes within and between assays was achieved, and the TBA of anti-Pfs25 control antibodies was highly reproducible.

**Conclusions:**

These results demonstrate a robust capacity to perform the SMFA in a medium-to-high throughput format, suitable for assessing large numbers of experimental samples of candidate antibodies or drugs.

## Background

Malaria is a prominent cause of morbidity and mortality in much of the world, with particularly devastating consequences for children and pregnant women in sub-Saharan Africa [1]. Malaria parasites are transmitted via the bite of infected female anopheline mosquitoes. While insecticide-treated nets, anti-malaria drugs, and indoor residual spraying of insecticides have together contributed to significant decreases in malaria incidence in many parts of Africa, the emergence and spread of drug-resistant parasites and insecticide-resistant mosquitoes are ever-present risks [2,3] that potentially threaten the recent gains in malaria control. It is now widely acknowledged that eliminating malaria from defined geographic areas will require additional tools [4]. The most effective vaccine-based strategy for malaria elimination would target both the pre-erythrocytic stage of the parasite life cycle to prevent infection, disease, and transmission of the parasite to mosquitoes, and the sexual erythrocytic and mosquito stages of the life cycle to prevent transmission, thus breaking the cycle of malaria transmission [4,5]. To develop a sexual-mosquito stage vaccine that interrupts malaria transmission (VIMT), antibodies are induced against promising target antigens and the resulting antisera are tested for transmission-blocking activity (TBA) using a standard membrane-feeding assay (SMFA) [6] in which test antisera are mixed with *Plasmodium* gametocytes cultured *in vitro* and fed to susceptible mosquitoes through an artificial membrane. The transmission-blocking activity (TBA) of test sera is calculated based on comparison of infection prevalence and intensity with that obtained in mosquitoes fed gametocytes mixed with control pre-immune serum.

While the SMFA is an essential tool for developing a sexual and mosquito stage VIMT, it is a labour-intensive, time consuming, and expensive assay that is subject to variability both within and between individual assays. To mass screen antibodies and drugs, a reliable, consistent and scalable SMFA is needed. To conduct industrial level SMFAs requires the continuous and reliable production of mature and highly infectious *Plasmodium falciparum* (Pf) gametocytes and healthy malaria-susceptible female *Anopheles* mosquitoes, infection of the mosquitoes by feeding them with gametocytes through an artificial membrane in the presence of negative and positive control sera, and assessing the mosquito infection levels by counting the number of oocyst stage parasites approximately one week after feeding.

In order to develop its *P. falciparum* sporozoite (SPZ)-based products, Sanaria has established industrial capabilities for production of *Anopheles stephensi* mosquitoes infected with the NF54 strain of *P. falciparum*, and systematic quantitative analysis of oocyst and sporozoite infections [7]. Exploiting these capabilities, Sanaria developed a robust, reliable, and consistent SMFA service to assess gametocytocidal and transmission blocking activity of drugs [8]. This service was subsequently scaled to undertake an industrialized screening of candidate transmission blocking serum samples for a commercial client. This paper demonstrates the successful assessment in duplicate of 188 serum samples for transmission-blocking (TB) activity in 74 independent SMFAs. The outcomes of these assays, demonstrating the feasibility of using the SMFA to reliably assess relatively large numbers of experimental samples are reported, and demonstrate that the patterns of infection are reproducible among assays.

## Methods

### Parasites

*Plasmodium falciparum* strain NF54 parasites, from Sanaria’s working cell bank, were cultured using human erythrocytes [8,9] in RPMI 1640 medium supplemented with human O^+^ serum and hypoxanthine. Gametocytogenesis was induced in blood stage parasites by maintaining the cultures with daily complete growth medium replacement and without the addition of fresh erythrocytes for 17–19 days. After 18 ± 1 (mean ± SD) days post induction, cultures were screened for use in SMFA based on abundance of mature Stage V gametocytes, exflagellation activity of microgametocytes and macrogametocyte: microgametocyte ratio.

### Mosquitoes

An *A. stephensi* strain SDA500 [10] colony was maintained in an insectary at 27 ± 1°C, 78 ± 5% RH, and a 12:12 light/dark cycle including 0.5 h dawn and dusk intervals. Larvae were fed a diet of Liquifry™ and Tetramin™ fish food. Adult mosquitoes were maintained in 30 × 30 × 30 cm cages, with sugar and water available *ad libitum*.

### Antibodies

Pre-immune sera and sera from placebo-vaccinated animals were used as negative controls for each SMFA. Positive control Pfs25 polyclonal antisera were obtained from MR4: TBV25H/2197 (MRA39), and TBV25H/2198 (MRA38). Both were deposited by Dr D C Kaslow [11]. MRA38 and MRA39 antisera were diluted 1:54 for use in SMFA. One-hundred and eighty-eight serum samples from mice immunized with Pfs25-based immunogens were obtained from Fraunhofer USA Center for Molecular Biotechnology (FhCMB) [12,13]. All sera were provided as blinded samples.

### Standard membrane feeding assay (SMFA)

#### Artificial blood meal preparation

Cultured stage V gametocytes were combined with uninfected human erythrocytes and serum to a final gametocyte concentration of 0.06-0.19%. For each SMFA, all blood meals were prepared from the same diluted gametocyte culture. The total volume of each blood meal was 270 μL, consisting of 150 μL of gametocytes diluted as above, plus 120 μL of serum (human O^+^ serum alone for negative control, antiserum against Pfs25 at a 1:54 final dilution in the blood meal for positive control or test sera of unknown samples). Use of Pfs25 antisera at 1:54 dilution was based on studies (data not shown) to identify an antibody concentration yielding significant reductions in both intensity and prevalence of oocyst stage (midgut) infections. For unknown test serum samples, 30 μL of test mouse serum was combined with 90 μL of human O^+^ serum and 150 μL of diluted gametocyte culture.

#### Mosquito feeding and maintenance

Thirty to 35 adult female *A. stephensi* mosquitoes were aspirated into a 450 mL cardboard container. The artificial blood meal maintained at 37°C was pipetted into a membrane feeding apparatus and presented to the mosquitoes through an artificial membrane. Each feeding apparatus was connected in series using rubber tubing and kept at approximately 37°C by water circulating through a 38°C water bath. Up to nine containers were fed simultaneously in one SMFA on individual meals containing negative and positive control sera, and up to six test mouse sera plus corresponding negative control mouse serum samples. Mosquitoes were allowed to feed at ambient temperature until all blood was consumed from the feeder, typically 20–30 minutes. Immediately after feeding, the mosquito containers were transferred to an incubator and thereafter maintained at 26 ± 1°C, 78 ± 5% RH, with a 12:12 light/dark cycle including 0.5 h dawn and dusk intervals, and supplied with sugar and water *ad libitum*.

#### Assessment of infection and transmission blocking activity

Six to nine days after feeding, 20–25 mosquitoes per container were dissected and midguts stained with 0.2% mercurochrome. Midguts were mounted on glass slides, and oocysts were counted using a compound microscope. Infection intensity (number of oocysts/mosquito) and infection prevalence (percentage of mosquitoes infected) were determined. TBA of positive control and experimental sera was calculated as a percentage reduction in either oocyst prevalence (TBA-P) or oocyst intensity (TBA-I) compared to the relevant control serum using the following formulae:

### Statistical analysis

**Figure 1:**
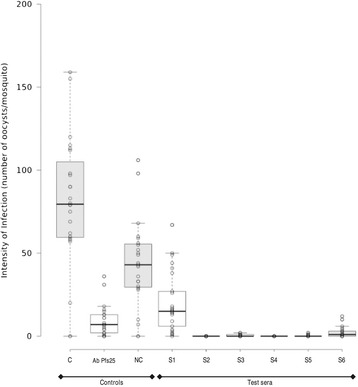
**Intensity of** ***Plasmodium falciparum*** **oocyst infections in a representative SMFA.** The results are shown for one of 74 SMFAs conducted for this study. Each point represents the number of oocysts per mosquito and the black bar indicates the geometric mean for each group. Boxes are 95% confidence intervals around the geometric means. C is non-immune control serum (O^+^ human serum). NC is negative control serum (pooled pre-immune mouse serum). S1 to S6 are serum samples from animals immunized with Pfs25 immunogens.

The data from 74 different feeding experiments of oocyst counts for all mosquitoes 12 - 27 mosquitoes per container) in the negative control and Pfs25 (positive control) groups were analysed in SAS 9.3. Calculation of the geometric means of oocysts numbers, percent prevalence of oocysts, range of oocyst counts, range of the positives for oocyst count, and sample size of mosquito midguts were calculated for both control and Pfs25 samples. The average of the geometric means was computed and 95% confidence intervals were calculated. Data in Figure 1 were plotted using BoxPlotR [14,15].

In order to explore the association between oocyst intensity and prevalence, the methods first described by Medley *et al.* [16] were applied. Several functional forms of the over-dispersion parameter (κ) were assessed and the best fitting model was chosen based on Akaike’s information criterion. Independent hyperbolic models were fitted to the data from the different treatments and control; 95% confidence bounds were estimated using 1,000 bootstrap samples.

## Results

### Reproducibility and robustness of SMFA

Seventy-four independent SMFAs were completed as described to test in duplicate the TBA of 188 unknown serum samples. Results of one typical SMFA are shown (Figure 1). Here, the TBA-I of the anti-Pfs25 MRA39 antibody (positive control) was 89% compared to the internal negative control and the TBA-I of the unknown serum samples ranged from 46 to 100% compared to the pre-immune pooled serum control.

The data from feeding positive and negative control sera were used to assess the reproducibility and robustness of the SMFA. For mosquito blood meals containing the negative control sera, there were robust and consistent oocyst infection intensities and prevalence rates (Table 1). In the 74 assay negative controls, in which mosquitoes received gametocytes with normal human serum, the geometric mean infection intensity was 46.1 oocysts/mosquito (range of positive samples 3.7-135.6), and mean infection prevalence was 94.2% (range 71.4-100.0%). Only four negative control feeds had geometric mean intensities less than ten oocysts/mosquito. A positive control was included in each SMFA: anti-Pfs25 rabbit antibodies MRA38 or MRA39 mixed with gametocytes. The 30 positive controls using MRA38 had a mean infection (oocyst) intensity of 1.3 (range 0–4.8), mean TBA-I of 97.0% (range 87.4-100%), and mean TBA-P of 63.5% (95% Cl = 53.3-73.7) (Table 1). Similarly for positive controls employing MRA39, the mean TBA-I was 81.6% (range = 36.5-98.9%) and the mean TBA-P was 12.9% (95% CI = 8.0-17.8) (Table 1). Although there was variability from assay to assay with respect to infection intensity in controls, there was little difference in either TBA-I or TBA-P between assays for the positive control samples (Table 1).

**Table 1:** **Summary of SMFA control results**

**Polyclonal anti-sera at 1:54 final dilution (number of assays)**		**Infection intensity (number of oocysts per mosquito)**			**Infection prevalence (percent of mosquitoes with oocysts)**		
		**Negative control**	**Pfs25 antibody**	**TBA-I**	**Negative control**	**Pfs25 antibody**	**TBA-P**
**MRA39 (n = 44)**	**Mean ± SD**	47.8 ± 32.9	8.4 ± 8.9	81.6 ± 12.9	93.8 ± 7.2	81.9 ± 16.5	12.9 ± 16.6
	**95% CI**	38.1, 57.5	5.8, 11.1	77.7, 85.4	91.7, 96.0	77.0, 86.8	8.0, 17.8
	**Range**	3.7, 135.6	0.1, 47.0	36.5, 98.9	71.4, 100.0	8.3, 100.0	−8.7, 89.6
**MRA38 (n = 30)**	**Mean ± SD**	43.7 ± 25.8	1.3 ± 1.6	97.0 ± 3.9	94.6 ± 4.4	34.6 ± 27.1	63.5 ± 28.6
	**95% CI**	34.5, 52.9	0.7, 1.8	95.6, 98.4	92.9, 96.3	24.9, 44.3	53.3, 73.7
	**Range**	7.1, 106.4	0.0, 4.8	87.4, 100.0	84.2, 100.0	0, 79.2	10.0, 100.0
**Pooled data (n = 74)**	**Mean ± SD**	46.1 ± 30.1			94.2 ± 6.3		
	**95% CI**	39.3, 52.5			92.7, 95.6		
	**Range**	3.7, 135.6			71.4, 100.0		

The positive and negative control data from 74 independent SMFAs are summarized to show the infection intensity and infection prevalence in mosquitoes fed negative control (human O^+^) serum or positive control anti-Pfs25 antibodies. Transmission blocking activities measured as effects on intensity (TBA-I) and prevalence (TBA-P) are calculated. In each SMFA there was one cage of negative control mosquitoes and one cage of positive control mosquitoes. For infection intensities, data are the arithmetic means ± standard deviation (SD) of the geometric mean intensities from each cage of negative or positive control mosquitoes as well as the 95% confidence intervals (CI) and range. For infection prevalence, data are the arithmetic means ± SD of the percent of mosquitoes infected in each cage of negative or positive control mosquitoes as wells as the 95% CI and range.

**Figure 2:**
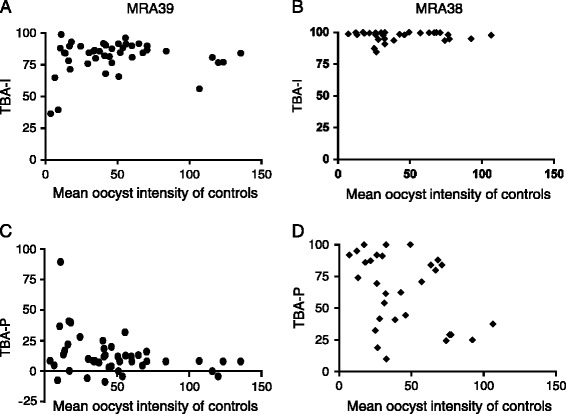
**Effect of blood meal quality on transmission blocking effect of two anti-Pfs25 monoclonal antibodies.** The relationship between infection intensity (geometric mean number of oocysts) in control mosquitoes and the infection intensity **(A, B)** and infection prevalence (proportion of mosquitoes with oocysts) **(C, D)** is plotted for mosquitoes fed anti-Pfs25 antibodies, MRA39 **(A, C)** and MRA38 **(B, D)** in the infectious blood meals.

The consistency of the TBA of the two positive control antisera in the 74 SMFAs was examined. The TBA-I and TBA-P were remarkably consistent for the MRA39 antiserum (Figure 2A, C), regardless of the numbers of oocysts per midgut in the respective control. The TBA-I was within the 70-100% range in 38 of the 44 assays (Figure 2A) across all oocyst intensities of the respective negative controls. However, reduction of prevalence rates with MRA39 was poor (Figure 2C). The TBA-P was consistently within the 0-30% range (40 of the 44 assays; negative values counted as 0%) (Figure 2C). There was a reduction in prevalence of greater than 75% in only one feeding event. Like MRA39, in all 30 SMFAs, TBA-I for the MRA38 antiserum ranged from 84 to 100% regardless of oocyst prevalence in the respective negative controls (Figure 2B). However, in contrast to MRA39, reductions in prevalence were more variable with MRA38, and TBA-P above 75% was achieved in 13 of the 30 SMFAs (Figure 2D). There was a slight but insignificant decline in TBA-P as oocysts numbers in the respective negative controls increased (Figure 2D).

### Transmission blocking activities of serum samples from mice immunized with Pfs25

**Figure 3:**
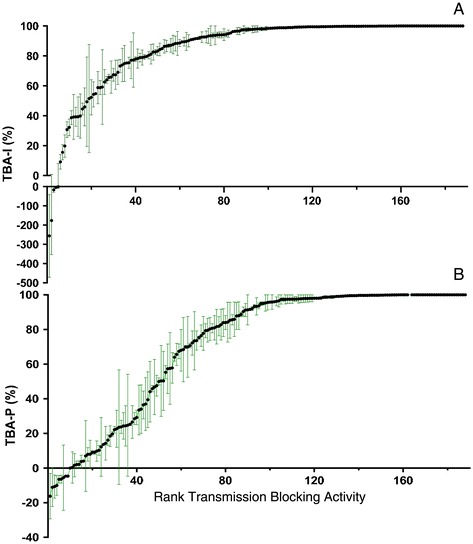
**Transmission blocking activity based on infection intensity of 188 unknown sera.** Samples are arranged in rank order of TBA. Each point indicates the arithmetic mean percent TBA of two independent assays and error bars represent the range of percent TBA. **(A)** TBA-I. Five sera increased infection intensity and 125 sera demonstrated >90% TBA-I. There was higher variability in TBA-I between duplicate assays in samples with <70% reduction in infection intensity. **(B)** TBA-P. Nine sera increased infection prevalence and 101 sera demonstrated >90% TBA-P. High variability in TBA-P between duplicate assays was observed only in sera with <80% reduction in prevalence.

One hundred and eighty eight unknown serum samples were tested, in duplicate, in 74 independently performed SMFAs (Figure 3). The TBA-I (Figure 3A) and TBA-P (Figure 3B) were reproducible between the duplicate assays for each sample when the percent inhibition was >75 and >80%, respectively. Below these values, the variability between assays was higher. Out of the 188 sera tested, five and nine serum samples enhanced oocyst infection intensity and infection prevalence, respectively, compared to the corresponding negative control (pre-immune) serum. Of the 188 sera tested, 31 sera demonstrated 100% TBA-I and 100% TBA-P, and 94 and 70 serum samples had TBA-I or TBA-P in the 90-99% range, respectively (Table 2).

**Table 2:** **Frequency distribution of reductions in intensity and prevalence of** ***P. falciparum*** **oocyst stage infections in** ***A. stephensi*** **for 188 serum samples tested in SMFA**

**Range of TBA**	**Number (%) of test sera with corresponding reduction in oocyst infection**	
	**Intensity (geometric mean oocysts/mosquito)**	**Prevalence (percent mosquitoes with oocysts)**
100%	31 (16.5%)	31 (16.5%)
90-99%	94 (50.0%)	70 (37.2%)
60-89%	38 (20.2%)	31 (16.5%)
30-59%	17 (9.0%)	16 (8.5%)
0-29%	3 (1.6%)	31 (16.5%)
<0%	5 (2.7%)	9 (4.8%)

### Relationship between oocyst intensity, oocyst prevalence, exposure to antibodies against Pfs25, and sample size

**Figure 4:**
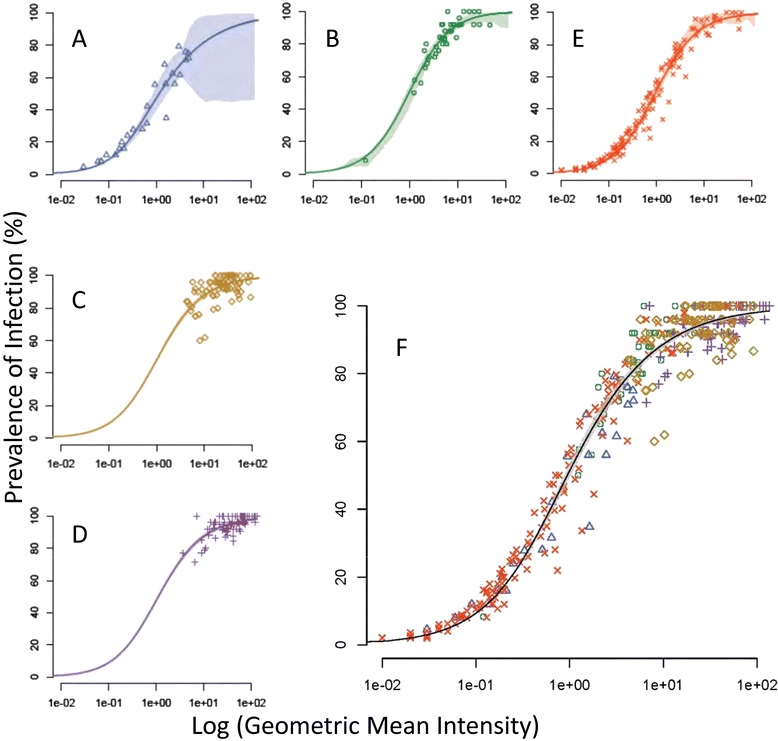
**Relationship between intensity and prevalence of** ***P. falciparum*** **oocysts in mosquitoes fed control monoclonal antibodies and test sera. (A)** MRA38 monoclonal antibody **(B)** MRA39 monoclonal antibody; **(C, D)** Negative control sera for (A) and (B) respectively; (**E)** Unknown experimental sera; **(F)** All data pooled to show consistency of relationship between the infection characteristics in all experiments. Shaded area is 95% confidence interval round the curve for the pooled data.

In order to demonstrate consistency of the SMFA regardless of infection intensity and prevalence in negative controls, the relationship between the log of the geometric mean oocyst intensity and the prevalence was examined separately for the two positive control antibodies (Figure 4A, B), their respective negative controls (Figure 4C, D) and for the test serum samples (Figure 4E). The data were first fitted using several functional forms of the over-dispersion parameter (κ) and the best fitting model, based on the lowest value for Akaike’s information criterion, was the hyperbolic model (Table 3). This was used to fit all data from different treatments and sample sizes.

**Table 3:** **Results of models fit to assess different functional forms of K, the overdispersion parameter** [16,31]

**Model**	**Functional form**	**Log likelihood**	**AIC**
Linear	*k*(*M*) = *αM* + *ε*	- 3204.317	6412.633
Power	*k*(*M*) = *αM* ^*β*^ + *ε*	- 3203.071	6412.142
Hyperbolic	$k(M)=\frac{\alpha M}{1+\gamma M}+\varepsilon$	- 3202.466	6410.931
Sigmoid	$k(M)=\frac{\alpha {M}^{\beta }}{1+\gamma {M}^{\beta }}+\varepsilon$	- 3202.357	6412.714

While all models fit the data similarly, the hyperbolic model was chosen as the final model based on its lowest Akaike’s information criterion (AIC).

Data from control mosquitoes and those exposed to Pfs25 antibodies fitted onto near identical curves (parameters for the hyperbolic model in each figure are given in Table 4) with negative controls clustered at the upper limit of the curve (Figure 4C, D) and those exposed to antibodies to Pfs25 (Figure 4A, B, E) scattered along the curve as intensity and prevalence were affected by the antibody treatments. The analysis confirmed the lesser effects of MRA39 (Figure 4B) *versus* MRA38 (Figure 4A) on prevalence and intensity of infection. Infection characteristics in mosquitoes fed MRA39 were more similar to controls, clustering to the upper right of the curve, whereas those fed MRA38 demonstrated a clear separation from controls and had consistently better reductions in prevalence rates and intensities. This pattern was mirrored in samples fed the experimental sera (Figure 4E). Here, the samples were blinded so it is possible that some samples were serum from placebo controls. Nevertheless, the various test samples were clearly distributed along the similar curves, demonstrating the robust nature of the SMFA and the persistence of the log-linear relationship. Surprisingly, there is little difference between treatments at low intensities and prevalence rates, but small differences appear at the higher infection rates. For each treatment, the 95% confidence bounds described by the model (shown as shaded areas around each curve) indicate that the model is an excellent fit to the data, with wider confidence bounds seen only where data associated with any treatment are sparse. For example, the wide 95% confidence bounds at the high end of the curve in Figure 4A and B are reflective of any data in these regions to allow model validation. The tight 95% confidence intervals for the fitted curves to the pooled data treatments (shaded area Figure 4F) reinforce the robust and reproducible nature of the assay based on the relationship between intensity and prevalence.

**Table 4:** **Parameter estimates from best fitting (hyperbolic) model**

**Treatment**	**α**	**γ**	**ε**
All data	−57. 1 (−57.6, −52.1)	12. 6 (8.0, 35.8)	5.3 (2.3-7.9)
1.	−0.10 (−60.6, 40.6)	1. 1 (−0.03, 44.6)	1.1 (−1.0, 12.0)
2.	−5. 5 (−25.5, 1.4)	5.1 (−0.24, 9.5)	1.7 (0.1, 5)
3.	−12.3 (−17.6, 0.6)	−105.3 (−600.5, 622.2)	0.8 (0.4, 0.9)
4.	−85.1 (−108.9, 0.4)	19.3 (−.02, 28.0)	5.4 (0.5, 7.4)
5.	−1.8 (−15.9, 0.9)	−248.1 (−344.4, 479.5)	0.8 (0.7, 1.0)

**Figure 5:**
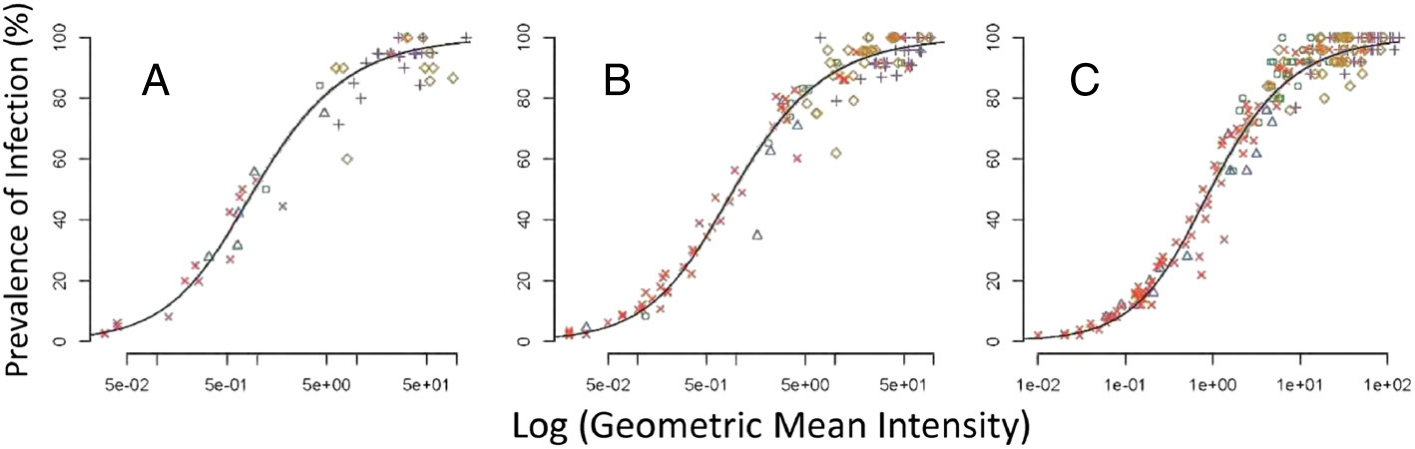
**Effect of sample size on the relationship between intensity and prevalence of** ***P. falciparum*** **oocysts in mosquitoes fed control monoclonal antibodies and test sera.** Experimental sera plus negative control serum and positive controls (MRA38 and MRA39) where were plotted against based on sample size of **(A)** ≤20 mosquitoes per sample (range = 14–20 mosquitoes per sample), **(B)** 21**–**24 mosquitoes per sample or **(C)** ≥25 mosquitoes per sample (range = 25–28 mosquitoes per sample). Symbols match those in Figure 4.

Evaluation of an SMFA to demonstrate its robustness and reproducibility is dependent upon the distribution of the data, which in turn govern the sample size required for assessment of TBA. The effects of sample size on fit to the model generated for the pooled data were examined (Figure 5). Regardless of sample size (<20 mosquitoes (Figure 5A), 21–25 mosquitoes (Figure 5B) or >25 mosquitoes (Figure 5C)), the samples fitted to the same log-linear distribution, although the distribution in samples of <20 mosquitoes were more scattered. However, it is clear that under the conditions described, increasing the numbers of mosquitoes assessed above 25 did not greatly increase the robustness and reproducibility of the assay.

## Discussion

Sexual erythrocytic and mosquito stage VIMTs are being developed with the expectation that they will play an important role in malaria elimination campaigns, and ultimately in malaria eradication. Such vaccine development has been carried out largely in research laboratories with limited capacity for scaling up the SMFA and for repeated throughput under highly controlled conditions, and often do not or cannot establish and maintain the rigorous quality control measures required of clinical laboratories or manufacturing facilities. By contrast, Sanaria routinely manufactures PfSPZ that meet all regulatory standards [9,17-25]. To do this Sanaria has established robust *P. falciparum* culture conditions and gametocyte production, coordinated with mosquito rearing and artificial membrane feeding methods that reproducibly yield highly infected *A. stephensi* mosquitoes. In order to monitor *P. falciparum* infections in mosquitoes Sanaria also routinely measures oocyst infection intensity and prevalence, and these assessments are performed and analysed by a team of trained staff members. Previous studies have developed medium to high throughput assays for mosquito and sexual stages of malaria that can be assessed *in vitro* [26] and a version of the SMFA that, after the feeding event, utilizes a *P. falciparum* strain derived from NF54 that has been genetically modified to express Green Fluorescent Protein-Luciferase for semi-automated downstream processing [27]. While such assays do provide higher throughput alternatives to the downstream (i.e. oocyst detection) elements of the SMFA, they are restricted to the genetically modified strain for assessment of transmission blocking effects. Consequently, the SMFA remains the gold standard for determining the effects of drugs or vaccines on transmission of multiple strains of *P. falciparum* from human host to mosquito vector. By exploiting its Good Manufacturing Practice (GMP) and Good Laboratory Practice (GLP) capabilities, Sanaria has developed an industrialized SMFA capacity to screen and evaluate SSM-VIMT and TB drug candidates that are in preclinical and clinical development, and can assess hundreds of samples in a relatively short period of time. This paper analyses the data generated from blinded SMFAs on 188 serum samples, performed semi-continuously, to conduct SMFA at an industrial scale with a high degree of reproducibility.

The efficacy of sexual erythrocytic and mosquito stage VIMTs [28], drugs [29], and other targeted interventions (e.g., [30]) are calculated based on percent reductions in infection (oocyst) intensity, infection (oocyst) prevalence or both. However, efficacy estimates from an assay system operated infrequently at widely spaced time intervals are subject to uncertainty from variables that impact each individual SMFA. These variables include oocyst intensity and prevalence in the control group, the number of experiments, and the number of mosquitoes analysed per experiment [31]. Moreover, it is inappropriate to calculate TBA-I from arithmetic means, though this has been used in previous studies [32,33], because oocyst infection intensity follows a negative binomial distribution at low intensities, but is closer to a Poisson distribution at high intensities [16,34,35]. Therefore, oocyst intensity should be presented as geometric mean if the data for *any* of the samples in the analysis are skewed, and TBA-I should be calculated based on geometric mean intensity of infection. In the present study the geometric mean oocyst intensities were therefore used to determine TBA-I for all samples.

While TBA of a given test sample is expressed typically as the percent reduction in oocyst infection intensity compared to a negative control [7,11,36], investigators have also analysed effects of antibodies or drugs on infection prevalence rates (TBA-P), measuring the proportion of fed mosquitoes exhibiting one or more oocysts [16,26,35] or any detectable sporozoites [37]. To provide a more complete picture of the effects of different TB treatments, both TBA-I and TBA-P oocyst infection data derived from dissections of 14–28 mosquitoes per sample are reported. As has been reported in previous studies [31], TBA based on measurement of TBA-I is greater than that based on measurement of TBA-P: 81.55% TBA-I *versus* 12.89% TBA-P for anti-Pfs25 antiserum MRA39, and 94.63% TBA-I *versus* 63.52% TBA-P for anti-Pfs25 antiserum MRA38. Within any given experiment, the consistent proportional reduction after Pfs25 antibody treatment compared to the respective controls indicates that the infectivity of the cultures was correlated for each independent experiment. Since the average number of oocysts in the control was not correlated with the percent reduction (Figure 2), this means that the dilution used (1:54 final dilution) of the control antibody contained sufficient activity to be able to quantify transmission reduction at even high control infection rates. Furthermore, the narrowness of these confidence intervals supports the reproducibility of results for this assay. The relationship between effects on infection intensity and prevalence is an important consideration in evaluating transmission-blocking interventions when the ultimate goal is to reduce prevalence rates to levels that can interrupt transmission in the field, in other words reduce the basic reproductive rate, R_0_, to a value less than 1 [38].

The dataset generated in this study is unique in terms of scale and consistency of method, and species and strains of parasite and vector. This consistency is reflected in the typically high reproducibility between results of duplicate feeding events (Figure 3) regardless of control infectivity and TBA-I or TBA-P. Duplicate rather than the typical triplicate assessments of a given sample were performed to allow higher throughput of the SMFA and was based on the high confidence in, and consistency of, the SMFA at Sanaria. Figure 3 now provides a powerful decision tool for future SMFAs at Sanaria. The occasional large difference between duplicate assays can be seen in the figure and will allow Sanaria to perform additional feeds on test samples where the difference between results is considered to be unacceptably high.

To conduct routine SMFAs requires the ability to continuously generate adequate numbers of highly infected mosquitoes. Sanaria has established a facility with fully trained staff executing procedures that allow continuous production of *A. stephensi* mosquitoes sustaining high *P. falciparum* oocyst infection prevalence rates and intensities. *A. stephensi* strain SDA500 mosquitoes and *P. falciparum* strain NF54 gametocytes are produced continuously and used to generate infected mosquitoes according to standard operating procedures described in a Biologics Master Files submitted to the US FDA. In 74 independently performed SMFAs the geometric mean oocyst infection intensity was 46.1 oocysts/mosquito and mean infection prevalence of 94.2% for negative controls (Table 1). Consistent infections are achieved through optimized and rigorously controlled mosquito colony maintenance procedures and gametocyte culture protocols that include quality control monitoring of stage V gametocytaemia, microgametocyte:macrogametocyte ratio and exflagellation activity.

Variable TBA outcomes can be problematic in SMFA. To address this, it is necessary to analyse a sufficient number of mosquitoes per treatment within a single SMFA to derive reproducible measurements of TBA. Examination of positive controls from 74 independent SMFAs using two different anti-Pfs25 antisera [39] demonstrated that feeding 30–35 mosquitoes and analysing a target 25 (actual range 14–28) of these mosquitoes for oocyst infection levels was sufficient to reliably measure TBA outcomes (Table 3, Figure 2A, B). Further, it is clear that even sample sizes below 20 mosquitoes can be sufficient to analyse the results under the highly controlled SMFA conditions described here. Using both prevalence and intensity in a fitted model provides the optimum use of all the available data rather than restricting analysis to a single parameter. It demonstrates the high consistency of the Sanaria’s SMFA and resulting mosquito infections, and enables pooling of subsets of data to be interrogated. Regardless of sample size, all data fitted to the robust intensity-prevalence relationship generated in Figure 4F. These findings are in contrast with those based on simulation models [33] and the *Plasmodium berghei* rodent parasite model [16], which suggested that using fewer than 50 mosquitoes per feed provided unrealistic TBA values. The reasons for this discrepancy are unclear, but likely reflect the greater reproducibility of SMFAs performed semi-continuously under good manufacturing (GxP)-like conditions with tightly controlled parameters.

A further important consideration to ensure that meaningful TBA assessments can be obtained from SMFA is to establish positive control antibody titres appropriate for the levels of oocyst intensity achieved in negative control assays. This was addressed in the present study by plotting TBA in positive controls against mean infection (oocyst) intensity and mean infection (oocyst) prevalence in the negative controls. At the antibody dilution used (1:54), the percent reduction in oocyst numbers (infection intensity) was similar for low and high numbers of oocysts in controls (Figure 2A, B). However, particularly for antibody, MRA38, there was an indication that as the oocyst prevalence increased in the controls, the percent reduction in oocyst numbers in mosquitoes fed the MRA38 antibody decreased (Figure 2D).

The *P. falciparum* SMFA is both time consuming and labour intensive. It requires the coordinated availability of *Anopheles* mosquitoes and infective gametocyte stage parasites. It is an expensive assay to perform, and for preclinical research and development is best carried out in a facility in which highly infected mosquitoes can be safely produced, maintained and manipulated. Nevertheless, it represents the best current assay for measuring the TB potential of candidate antibodies, drugs or other targeted interventions. The current study represents, as far as can be ascertained, the first report of an industrial scale application of the SMFA to a large number of TB experimental samples conducted semi-continuously and highly reproducibly over an extended time period.

## Conclusions

By focusing upon consistency of *P. falciparum* gametocyte production, *A. stephensi* mosquito production, infectious blood meal characteristics and feeding, and subsequent maintenance of infected mosquitoes consistent with good manufacturing practices (GMP). Sanaria has not only standardized the SMFA but progressed it to a medium-throughput system in which the TBAs of many unknown samples can be assessed. Additionally, by including both intensity and prevalence in the same model-fitting analysis, maximum use of the data is made in a statistically robust way that incorporates control data integrated into every feeding event.

## Abbreviations

CI: Confidence interval
FDA: Food and Drug Administration
GMP: Good Manufacturing Practices
h: hour(s)
P: *Plasmodium*
Pf: *Plasmodium falciparum*
Pfs25: *Plasmodium falciparum* 25 kiloDalton surface molecule
PfSPZ: *Plasmodium falciparum* sporozoite
R_0_: Basic reproductive rate or number
RH: Relative humidity
SMFA: Standard membrane feeding assay
TB: Transmission-blocking
TBA: Transmission-blocking activity
TBA-I: Transmission-blocking activity calculated using infection intensity
TBA-P: Transmission-blocking activity calculated using infection prevalence
VIMT: Vaccine that interrupts malaria transmission

## References

[1] WHO/Global Malaria Programme (2013). World Malaria Report 2013.

[2] Maxmen A (2012). Malaria surge feared. Nature.

[3] Alonso PL, Tanner M (2013). Public health challenges and prospects for malaria control and elimination. Nat Med.

[4] Alonso PL, Ballou R, Brown G, Chitnis C, Loucq C, Moorthy V (2011). A research agenda for malaria eradication: vaccines. PLoS Med.

[5] Plowe CV, Alonso P, Hoffman SL (2009). The potential role of vaccines in the elimination of falciparum malaria and the eventual eradication of malaria. J Infect Dis.

[6] Duffy PE, Kaslow DC (1997). A novel malaria protein, Pfs28, and Pfs25 are genetically linked and synergistic as falciparum malaria transmission-blocking vaccines. Infect Immun.

[7] Lensen A, van Druten J, Bolmer M, van Gemert G, Eling W, Sauerwein R (1996). Measurement by membrane feeding of reduction in *Plasmodium falciparum* transmission induced by endemic sera. Trans R Soc Trop Med Hyg.

[8] Adjalley SH, Johnston GL, Li T, Eastman RT, Ekland EH, Eappen AG (2011). Quantitative assessment of *Plasmodium falciparum* sexual development reveals potent transmission-blocking activity by methylene blue. Proc Natl Acad Sci U S A.

[9] Hoffman SL, Billingsley P, James E, Richman A, Loyevsky M, Li T (2010). Development of a metabolically active, non-replicating sporozoite vaccine to prevent *Plasmodium falciparum* malaria. Hum Vaccin.

[10] Feldmann AM, Ponnudurai T (1989). *Anopheles stephensi* for refractoriness and susceptibility to *Plasmodium falciparum*. Med Vet Entomol.

[11] Stowers AW, Keister DB, Muratova O, Kaslow DC (2000). A region of *Plasmodium falciparum* antigen Pfs25 that is the target of highly potent transmission-blocking antibodies. Infect Immun.

[12] Jones RM, Chichester JA, Mett V, Jaje J, Tottey S, Manceva S,C (2013). A plant-produced Pfs25 VLP malaria vaccine candidate induces persistent transmission blocking antibodies against *Plasmodium falciparum* in immunized mice. PLoS One.

[13] Jones RM, Chichester JA, Manceva S, Gibbs SK, Musiychuk K, Shamloul M (2015). A novel plant-produced Pfs25 fusion subunit vaccine induces long-lasting transmission blocking antibody responses. Hum Vaccin Immunother.

[14] Spitzer M, Wildenhain J, Rappsilber J, Tyers M (2014). BoxPlotR: a web tool for generation of box plots. Nat Methods.

[15] BoxPlotR: a web-tool for generation of box plots. http://boxplot.tyerslab.com/. Assessed 20 May 2014. 10.1038/nmeth.2811 PMC3930876 24481215

[16] Medley GF, Sinden RE, Fleck S, Billingsley PF, Tirawanchai N, Rodriguez MH (1993). Heterogeneity in patterns of malarial oocyst infections in the mosquito vector. Parasitology.

[17] Lyke KE, Laurens M, Adams M, Billingsley PF, Richman A, Loyevsky M (2010). *Plasmodium falciparum* malaria challenge by the bite of aseptic *Anopheles stephensi* mosquitoes: results of a randomized infectivity trial. PLoS One.

[18] Epstein JE, Tewari K, Lyke KE, Sim BK, Billingsley PF, Laurens MB (2011). Live attenuated malaria vaccine designed to protect through hepatic CD8 + T cell immunity. Science.

[19] Laurens MB, Billingsley P, Richman A, Eappen AG, Adams M, Li T (2013). Successful human infection with *P. falciparum* using three aseptic *Anopheles stephensi* mosquitoes: A new model for controlled human malaria infection. PLoS One.

[20] Roestenberg M, Bijker EM, Sim BK, Billingsley PF, James ER, Bastiaens GJ (2013). Controlled human malaria infections by intradermal injection of cryopreserved *Plasmodium falciparum* sporozoites. Am J Trop Med Hyg.

[21] Seder RA, Chang LJ, Enama ME, Zephir KL, Sarwar UN, Gordon IJ (2013). Protection against malaria by intravenous immunization with a nonreplicating sporozoite vaccine. Science.

[22] Sheehy SH, Spencer AJ, Douglas AD, Sim BK, Longley RJ, Edwards NJ (2013). Optimising controlled human malaria infection studies using cryopreserved parasites administered by needle and syringe. PLoS One.

[23] Shekalaghe S, Rutaihwa M, Billingsley PF, Chemba M, Daubenberger CA, James ER (2014). Controlled human malaria infection of Tanzanians by intradermal injection of aseptic, purified, cryopreserved Plasmodium falciparum sporozoites. Am J Trop Med Hyg..

[24] Hodgson SH, Juma EA, Salim A, Magiri C, Kimani D, Njenga D (2014). Evaluating Controlled Human Malaria Infection in Kenyan Adults with Varying Degrees of Prior Exposure to Plasmodium falciparum using sporozoites administered by intramuscular injection. Frontiers in Microbiology.

[25] Mordmüller B, Supan C, Sim KL, Gómez-Pérez GP, Salazar CLO, Held J (2015). Direct venous inoculation of Plasmodium falciparum sporozoites for controlled human malaria infection: a dose-finding trial in two centres. Malaria J.

[26] Bolscher JM, Koolen KM, van Gemert GJ, van de Vegte-Bolmer MG, Bousema T, Leroy D, et al. A combination of new screening assays for prioritization of transmission-blocking antimalarials reveals distinct dynamics of marketed and experimental drugs. J Antimicrob Chemother. 2015; dkv003. [Epub ahead of print]. 10.1093/jac/dkv003 25667405

[27] Stone WJ, Churcher TS, Graumans W, van Gemert GJ, Vos MW, Lanke KH (2014). A scalable assessment of *Plasmodium falciparum* transmission in the standard membrane-feeding assay, using transgenic parasites expressing green fluorescent protein-luciferase. J Infect Dis.

[28] Carter R (2001). Transmission blocking malaria vaccines. Vaccine.

[29] Delves MJ (2012). Plasmodium cell biology should inform strategies used in the development of antimalarial transmission-blocking drugs. Future Med Chem.

[30] Dinglasan RR, Fields I, Shahabuddin M, Azad AF, Sacci JB (2003). Monoclonal antibody MG96 completely blocks *Plasmodium yoelii* development in *Anopheles stephensi*. Infect Immun.

[31] Churcher TS, Blagborough AM, Delves M, Ramakrishnan C, Kapulu MC, Williams AR (2012). Measuring the blockade of malaria transmission–an analysis of the Standard Membrane Feeding Assay. Int J Parasitol.

[32] van der Kolk M, De Vlas SJ, Saul A, van de Vegte-Bolmer M, Eling WM, Sauerwein RW (2005). Evaluation of the standard membrane feeding assay (SMFA) for the determination of malaria transmission-reducing activity using empirical data. Parasitology.

[33] Miura K, Deng B, Tullo G, Diouf A, Moretz SE, Locke E (2013). Qualification of standard membrane-feeding assay with *Plasmodium falciparum* malaria and potential improvements for future assays. PLoS One.

[34] Ichimori K, Curtis CF, Targett GA (1990). The effects of chloroquine on the infectivity of chloroquine-sensitive and -resistant populations of *Plasmodium yoelii* nigeriensis to mosquitoes. Parasitology.

[35] Billingsley PF, Medley GF, Charlwood JD, Sinden RE (1994). Patterns of infection of *Plasmodium falciparum* in wild caught *Anopheles* mosquitoes. Am J Trop Med Hyg.

[36] Peiris JS, Premawansa S, Ranawaka MB, Udagama PV, Munasinghe YD, Nanayakkara MV (1988). Monoclonal and polyclonal antibodies both block and enhance transmission of human *Plasmodium vivax* malaria. Am J Trop Med Hyg.

[37] Boudin C, Lyannaz J, Bosseno MF, Carnevale P, Ambroise Thomas P (1991). Epidemiology of *Plasmodium falciparum* in a rice field and a savanna area in Burkina Faso: seasonal fluctuations of gametocytaemia and malarial infectivity. Ann Trop Med Parasitol.

[38] Chiyaka C, Tatem AJ, Cohen JM, Gething PW, Johnston G, Gosling R (2013). Infectious disease. The stability of malaria elimination. Science.

[39] Kaslow DC, Bathurst IC, Lensen T, Ponnudurai T, Barr PJ, Keister DB (1994). *Saccharomyces cerevisiae* recombinant Pfs25 adsorbed to alum elicits antibodies that block transmission of *Plasmodium falciparum*. Infect Immun.

